# Abortion, emotions, and health provision: Explaining health care professionals' willingness to provide abortion care using affect theory

**DOI:** 10.1016/j.wsif.2018.09.002

**Published:** 2018

**Authors:** Deirdre Niamh Duffy, Claire Pierson, Caroline Myerscough, Diane Urquhart, Lindsey Earner-Byrne

**Affiliations:** a Manchester Metropolitan University, United Kingdom of Great Britain and Northern Ireland; b University of Liverpool, United Kingdom of Great Britain and Northern Ireland; c Edge Hill University, United Kingdom of Great Britain and Northern Ireland; d University College Dublin, Republic of Ireland

## Introduction

This article interrogates health care professionals' (HCPs) decision to withdraw from or provide abortion care using cultural theories of affect and emotion. It argues that emotional reactions to imagined futures influence the actions of health care practitioners. This proposition draws together cultural theory of affect with qualitative evidence from a scoping study of abortion care and Ireland. Our analysis suggests that discussions as to why care is or is not provided need to look at the emotional entanglements and affective feel of (written) legal frameworks from the position of the subjects of those frameworks as well as the frameworks themselves. In doing so, our interrogation provides critical insight for considerations of abortion access in the Irish context and globally. This is an important contribution given the concurrent global debates about why abortion care is or is not provided and how to ensure abortion is completely accessible.

Our analysis has particular significance in the Irish context. Abortion law and policy on the island of Ireland is in flux (Mishtal, 2017). Campaigns against impediments to access abortion in the Republic of Ireland, Northern Ireland, and abroad have both drawn attention to and emboldened challenges of the existing hyperrestrictive (De Londras, 2017) legal regimes in both jurisdictions on the island. In February 2018, following the recommendations of a Citizens' Assembly and cross-party Joint Oireachtas (parliament) Committee, the Republic of Ireland's Government committed to holding a referendum on repealing Article 40.3.3 of the constitution. This article – instituted as the 8th Amendment by popular referendum in 1983 – prohibits abortion by committing the state to protecting the ‘right to life’ of the ‘unborn’ (for more detailed legal analysis see: Enright & De Londras, 2018). The provision of information about abortion is restricted by the 1995 Regulation of Information (Services Outside the State for the Termination of Pregnancies) Act (known as the Abortion Information Act). Referral for abortion under health grounds is tightly controlled by this legislation and the 2015 Protection of Life During Pregnancy Act. A proposal to repeal the 8th Amendment (and associated Amendments) was supported by two-thirds of the electorate in a referendum in May 2018 and the government has committed to implementing new, more liberal, abortion law by 2019. However, this legislation has yet to be debated in Oireachtas and the exact implementation schedule and process is still unclear.

In Northern Ireland, access to abortion has been emphasised as a core issue during recent elections to the Northern Ireland Assembly (Pierson and Bloomer, 2017). Although part of the United Kingdom, the 1967 British Abortion Act has never been adopted in the jurisdiction and abortion is regulated by the 1861 Offences Against the Person Act (ss.58–59). Like women living in the Republic, women living in Northern Ireland have extremely limited abortion access. While case law following *R v Bourne* ([1939] 1 KB 687) allows for abortion for the purposes of preventing a woman becoming “a mental and physical wreck”, the circumstances under which abortion is permissible has never been clearly defined and the majority of women seeking abortion travel outside the country or import ‘abortion pills’ illegally (Aiken, Gomperts, & Trussell, 2017). Members of the UK Parliament at Westminster have expressed the opinion that abortion needs to be made accessible to women living in Northern Ireland. Lobbying by pro-choice groups and a recent Private Members' Bill by Stella Creasy (Labour MP for Wallasey) have led to the introduction of financial support for women resident in Northern Ireland compelled to travel to England for abortions. Influenced by the referendum in the Republic, there has also been substantial movement (by Creasy and activists) towards the decriminalisation of abortion across the UK (through the repeal of the 1861 Offences Against the Person Act). A strong narrative within this campaign is the need to remove the legal grounds for Northern Ireland's prohibitive abortion policy.

The objective of liberalisation campaigns is to make abortion more accessible. However, this article argues that the link between liberalisation in law and policy as written and increased accessibility is complex and legal change is only the first stage in this process. As research in contexts where abortion is legally permissible has shown, there is not a straightforward relationship between legality and accessibility (De Zordo & Mishtal, 2011; Sheldon, 2016). Sexual and reproductive health research (Jain, 1992; Bloomer & Hoggart, 2012; Pierson and Bloomer, 2017) emphasises that access is socially and culturally contingent. It requires not just the availability of legal abortion but the provision of abortion (by health care practitioners) and the request for abortion (by patients).

Provision is the focus of this article. Using evidence from interviews with health care practitioners in Ireland (Ireland and Irish will be used to indicate both jurisdictions unless otherwise stated) we argue that the reason they do or do not provide abortion care cannot be solely explained as a reaction to provisions of law or policy as *written*. Their practices are also influenced by emotions and future-thinking related to these laws – the *feel* of the law. Of particular significance, we argue, are the feelings evoked by *professional* futures (where a HCP is judged to have acted outside the law) and by *patients*' futures (if particular forms of care is made unavailable to them). As pro-choice activists during the referendum campaign in the Republic of Ireland noted, we cannot assume that *access* to abortion will automatically result from *legalisation* of abortion (the fact that access is also an economic issue was synthesised in their slogan – *free*, *safe*, *legal*). A focused discussion how provision of abortion is regulated by forces outside of, but entangled with, legal frameworks is therefore of critical importance.

These arguments are not just significant in the Irish context. They provide insight for global debates about the regulation of HCPs and the reasons why abortion care is or is not provided. Given on-going debates about the limits – legal, structural, political and cultural – on abortion access our analysis makes an important contribution. Our findings are also significant given the dearth of evidence from the perspectives of HCPs. Health providers' voices are significantly under-represented in research on abortion and sexual and reproductive health (Britton, Mercier, Buchbinder, & Bryant, 2017). The activities and experiences of HCPs in restrictive abortion regimes has only recently become a prominent subject of discussion in writing on abortion and reproductive health.

The article is developed in three stages. First, we will introduce the conceptual frame underpinning our analysis and why is it important to our study. Second, we will interrogate findings from interviews with HCPs conducted as part of a Wellcome Trust-funded study on abortion care and Ireland. Third, and finally, we will discuss what our findings and analysis mean for efforts to make abortion accessible.

## Affect, HCPs, and abortion care practice

Literature on abortion has highlighted that HCPs decisions on what care to provide and what activities to engage in is frequently based on cultural and social discourses around abortion and patienthood (De Zordo, 2017; De Zordo & Mishtal, 2011; Purcell, Cameron, Lawton, Glasier, & Harden, 2017) rather than policy or legislation. Particular attention is paid to issues of conscientious objection (De Zordo & Mishtal, 2011), religion (Mishtal, 2017), and the effect of abortion stigma (Beynon-Jones, 2013, Beynon-Jones, 2017; Kumar, Hessini, & Mitchell, 2009) on the decision by HCPs to engage in or withdraw from certain practices. Into this burgeoning debate, this article will interject a further analysis of what directs HCPs activities in restrictive abortion regimes - their fear of their own and their patients' future.

Activists and commentators in Ireland have already drawn attention to fear and emotion – ‘chilling effects’ – as entangled with abortion law and abortion care in Ireland (Enright & De Londras, 2018). HCPs addressing the 2017–18 Joint Oireachtas Committee on abortion reform in the Republic of Ireland including the Master of the National Maternity Hospital, Dr. Rhona Mahoney, and the Irish Association of General Practitioners (IAGP), spoke fluidly about HCPs fears. These ‘chilling effects’ are influenced by restrictive legislation. However, the interplay between fear, health care practice, and law are complex. HCPs decisions to offer care or not should be more extensively interrogated. To do this we will draw on affect theory and the dynamics of regulation.

A core argument of theorists of affect is that the subjects' imagining of the future orientates their actions in the present. Ahmed (2010) discusses this through an analysis of happiness, suggesting that the future experience of happiness - regardless of the conditions of this happiness - regulates subjects. This is resonant with Deleuzean models of regulation and subjectivity and the effect of the ‘not yet formed’ on the ‘now’ (Deleuze & Boundas, 1991). Deleuze argues that subjects' actions become ossified as they connect (either in thought or in sensation or both) with what he calls the still-emergent Body without Organs or BwO (Bignall, 2010; Deleuze & Boundas, 1991).

According to affect, the regulation of the subject by means of an imagined futurity is reflected - and achieved - through how the subject *feels* in the present when they think about a future. This includes both emotions (e.g. hope, fear, despair, anger) and sensations (e.g. disgust, discomfort, desire). As well as Ahmed's analysis of affective regulation through happiness and optimism, Berlant (2011) proposes that feelings of despair experienced when a desire is felt as unattainable have an equally strong influence over subjects' actions. In the context of social policy and practice, writing on the effect of future judgements and evaluations, Sellar (2015) and Hood (2006) highlight how fear and dread about what could happen in the future effects the actions of professionals in education (Sellar) and health and social care (Hood). Similar arguments are made by Pykett (2015) and Hunter (2015), the latter of whom argues that UK governing elites increasingly harness feelings about what will happen in the (as yet indeterminate) future to orientate subjects towards or away from particular behaviours. Notably, Pykett and Hunter underline the fact that, in terms of directing subjects' actions in situ, what legal frameworks or policy actually say about particular courses of action matter less than subjects' feelings towards these courses of action (or rather, what they feel will result from not following them). This is not to say that law and policy do not impact subjects' decision-making but that decision-making is not restricted by law as written alone. In simpler terms, affect argues that people refrain from doing things not just because laws say they should not do them; they refrain from doing things because of what they feel when they imagine what will happen should they continue doing them.

Combined then, writing on affect and governing, proposes that actions and sensations by subjects are not only reflective of law and policy as written. They are also reflective of the imposition - which Berlant (2011) describes as ‘suspension’ - of an as-yet-unrealised future on the subject. Significantly, while writers such as Ahmed and Berlant predominantly discuss this dynamic in terms of the *control* or restriction of the subject, this is not necessarily the only response by subjects when the BwO is felt or the future is sensed at an emotional level. What is notable about affect perspectives, and what differentiates them from other writing on the orientation of the subject through sensings and intensity - particularly Foucault's arguments on surveillance and discipline - is that the orientation of the subject through the suspension of an *imagined* future is inherently ambivalent. What marks these instances, as Massumi (2002) highlights, is their generativity, which can result in both control and self-restriction and intense creativity and progression (Duffy, 2018). This duality is explored in depth by Gregg and Seigworth (2010) who describe affective moments and the crossing over of present reality and potential futurity as a space of openness which facilitates both control and innovation. Gregg and Seigworth (2010) describe the subjects' response to the imagining of futures in the present as both “sticky” and “stretchy” (Gregg & Seigworth, 2010: 14). It is a moment which simultaneously restricts subjects according to what the futurity is imagined to involve and opens up possibilities for this futurity to be something else.

In terms of HCPs and questions of abortion access, affect is then a particularly useful frame as it also explains why fear of future is not debilitating (in the sense that no care is provided) but productive. Care is provided by HCPs but that care may be *different* to what is written in law and policy either in its creativity (engaging in practices not anticipated) or its conservativism (withdrawing from practices permissible or even promoted by law and policy). To demonstrate this we will now look at qualitative evidence from HCPs based in Ireland.

## Methodology

This article uses evidence from an interdisciplinary exploratory study of the Liverpool-Ireland Abortion Corridor, one of the Irish ‘abortion trails’. Irish experiences of abortion have and continue to be typified by travel across borders, most usually to England. The regularity of Irish women travelling elsewhere for abortion services has led to the emergence of ‘abortion trails’ (Rossiter, 2009). These trails have cultural and political as well as practical significance. Pro-choice groups such as Speaking of I.M.E.L.D.A. (Ireland Making England the Legal Destination for Abortion) and the Abortion Rights Campaign have pointed to the abortion trails as emblematic of Ireland outsourcing abortion to other countries (Enright, 2014; Fletcher, 2015). Liverpool, which had been a destination for women seeking adoption services since the late 19th century (Earner-Byrne, 2003), became a key location for women looking for abortions after the 1967 Abortion Act. However, the research team noted that the abortion trails had not been fully examined from a healthcare perspective. Literature had focused on issues of rights, medical tourism, pro-choice activism, and stigma. The focus of the LIAC study was twofold: (i) the dynamics of an ‘abortion trail’ and (ii) the impact of the need to travel outside Ireland for abortion on care-giving and the structure of care.

The LIAC research was designed as a single case scoping study of abortion travel and care. Single case study research is a useful mechanism for interrogating complex phenomena in depth (Flyvbjerg, 2006; Yin, 2009). The central proposition of case study research is that ‘context-dependent knowledge’ is as - if not more - valuable as ‘context independent’ knowledge (Yin, 2009). In-depth single case study can provide insight to stimulate further research and for complex problems can be more useful than broader, less in-depth information.

The research used archival documentary evidence (from public and private archives in Liverpool and the island of Ireland) and qualitative interview data. A total of 36 interviews with two different groups (activists and health care professionals) were conducted in England, Northern Ireland, and the Republic of Ireland between July and August 2016 by two researchers. This article uses data gathered through face-to-face and Skype interviews with HCPs based in Ireland. Participants were contacted through a combination of purposive sampling and via gatekeepers working in healthcare in Northern Ireland and the Republic. Key organisations were identified through desktop and archival research and contacted via email. Respondents to the initial request for participants were invited to circulate the invitation and details of the study to colleagues. Interviewees self-selected and were not asked about their personal perspectives on abortion or abortion law or the effect of these on their practices directly. Interviews were semi-structured, with participants encouraged to include information they felt relevant to the study. Participants were also invited to access verbatim interview transcripts and to make redactions or additions they felt appropriate. No participant asked for material to be added or redacted. This finalised transcript was then used as the basis for analysis. The final sample (n = 19) including participants based in Northern Ireland (n = 6) and the Republic of Ireland (n = 11).

As the objective of the study was to gain insight into discourses and dynamics within the care system as a whole, recruitment did not focus on one specific discipline or sector of the health workforce. As Nancarrow and Borthwick (2005) have noted, the boundaries between specific health professions have become increasingly fluid since the 1980s. This is reflected in research on abortion practice focused on the lived experience of legal regulation and stigma which include perspectives of a range of different HCPs (De Zordo & Mishtal, 2011; Diniz, Madeiro, & Rosas, 2014; Purcell et al., 2017). Our sample included clinicians (n = 10), family planning and sexual and reproductive health counsellors and advisory agencies (n = 6), and abortion clinic and charity managers (n = 3). A further 17 activists were interviewed but that data is not included in analysis for this article. One clinic manager and the CEO of a charity providing financial assistance in England were also interviewed as part of the research.

Interviews were transcribed verbatim by the researchers and data was coded using NVIVO 10 software by two researchers and subjected to thematic analysis. Thematic analysis is a method for identifying, analysing and reporting patterns (themes) in data (Braun & Clarke, 2006). Two researchers (Duffy, 2018 and Pierson and Bloomer, 2017) read and conducted early analysis on transcripts separately. They then came together to construct a framework of initial codes. This framework was then applied to transcripts to establish themes and sub-themes. These themes and sub-themes were combined with emerging literature on abortion care and abortion to identify areas for discussion and further research raised by the research.

Ethical approvals for the research were provided by Manchester Metropolitan University (Cheshire) Research Ethics and Governance Committee.

## Findings

While the study's initial focus was care and abortion travel, a significant theme in the data was the everyday practices of HCPs in Ireland and how HCPs' perceived these to be impacted by abortion laws in both jurisdictions. Qualitative data from the study offered significant insight into how HCPs' actions were influenced by a visceral reaction to what would result from action or inaction. Theoretically this spoke to writing on governing as an affective process where subjects' were oriented towards or away from particular actions by the manipulation of emotional registers associated with imagined futures (Pykett, 2015; Hunter, 2015; Ahmed, 2010; Duffy, 2018). This section will illustrate how evidence from the LIAC study speaks to the affective governing of HCPs practices in the context of abortion through focusing on the dominant emotion reflected in interview data - fear. Principally, it will illustrate how HCPs' fear of imagined futures - their own and that of abortion seekers - influences HCPs practices. These fears were frequently associated with overly restrictive interpretations of law and policy. Furthermore, using examples from the LIAC study participants, it will indicate that the response of HCPs to fear of future is not just to withdraw from certain practices but to experiment with and think creatively about how to deliver care.

### Fears of personal futures

Interviewees in each category and jurisdiction spoke extensively about the impact of fear of their own future (particularly future prosecution) on their actions. The reflected from an individual and collective perspective. Participants in the LIAC study suggested that the provision of information about abortion is particularly negatively affected by fears of imagined futures. In Northern Ireland, one interviewee (a midwife and a representative of a professional midwifery organisation) described midwives, particularly front-line staff in hospitals, as “petrified of giving any information which may be construed as advice, that might be interpreted as advocating or assisting a women in procuring an abortion” (Interview 15). The use of the word ‘might’ is significant here. The argument being expressed by this interviewee is not necessary that their actions are illegal but whether the interpretation of their actions could lead to prosecution at a later point.

Similar comments were made by another interviewee (a representative of a professional nursing organisation) who highlighted the ambiguity of terminology regarding what constituted illegal behaviour in recent guidance from the Northern Ireland Department of Health regarding the legal status of abortion care (in 2013 and 2015). While the latter, this interviewee stated, was slightly clearer in terms of what was and was not illegal than the former, he argued that members of his organisation still felt like they “are being asked to interpret the law” (Interview 5) without any specific guidance on how to do this. Like the representative of a professional midwifery organisation, he spoke about practitioners' fear of providing abortion care stating that:

*I do feel that nurses are really scared, if they are seen to be pointing a women in a particular direction, particularly if that involved accessing information on termination obviously outside of NI that they are putting themselves in a serious position with regard to the law.*

(Interview 5)

Again this interviewee describes a situation where the main fear is not illegal provision of information but whether providing such information will be judged as illegal in the future and the outcomes of this future interpretation.

Fear of future also emerged in interviews in the Republic of Ireland particularly in relation to the provision of information about abortion to women. Under the terms of the Regulation of Information (Services outside the State for termination of pregnancy) Act 1995, health professionals can provide women with information about abortion care as long as they provide information about continuation of pregnancy and adoption at the same time (a ‘three options’ approach). The premise of this legislation is that, while information should be made available, abortion is not to be *promoted* by health practitioners. Although a number of interviewees initially argued that they were comfortable with providing information about abortion to patients, interview data revealed fearfulness of the potential for legal behaviour to be interpreted and framed as illegal at a future point by opponents to abortion care. Interviewees spoke of how health professionals – in hospitals and in family planning agencies – had been recorded covertly during conversations about pregnant women's options under the law. These covert recordings were later edited and (falsely) presented as exemplary of health professionals breaking abortion information law. As one interviewee, an obstetric specialist, described:

*I mean I think one of the difficulties is that, you know, if you assist somebody then that's a criminal offense. And, for us, these are our livelihoods so that criminal offense would mean a loss of our profession and our income and everything we've worked for the last twenty, twenty-five years. And there have been cases of people masquerading as patients, going to clinics, and then recording them, and then turning on the nine o'clock news and, there you are, giving advice to someone – not me now – but these recordings have been released to the media. And so that…environment is there. So you're counselling somebody and they have a number of people with them and they could be recording consultations. That's becoming more and more common now. They're not obligated to tell you and so I think one has to be careful that you don't have somebody, as I say that has happened as it has gone onto the media. People have been taped inadvertently and then released.*

(Interview 26)

HCPs working in family planning organisations as pregnancy and crisis pregnancy counsellors provided similar accounts. One spoke of the need to be careful “who was in the room” (Interview 23), while others described how they had been reported to the Garda Síochana (the police) as promoting abortion based on an edited covert recording.

What is interesting about these accounts from an affect theory perspective is that HCPs are not afraid of whether their behaviour is illegal but whether it can or will be interpreted as illegal *in the future* and the result of this. The main fear of interviewees in both jurisdictions was not necessarily whether they were being recorded or not, it was how this recording could be edited and to who it could be presented at a future point. Decision-making is complex and influenced by the law, its potential interpretation, and HCPs' emotional feelings towards the future that would result from how the law could be interpreted.

It was not just the potential for future prosecution that resulted in fearfulness and self-regulation, it was also fear of their future professional status. Interviewees spoke of how they could be struck off if they were judged as engaging in unprofessional or illegal practices. Interestingly, some interviewees stated that, as disclosures of confidential patient-HCP conversations was also prohibited there was a tension between their fears of prosecution for illegal provision of information/promotion of abortion and their fears for being later judged as having breached professional codes of conduct. This concern is illustrated in the following quote:

*Under the NMC's code you are required to protect confidentiality but that has limits and crime can override that duty. It is unfair that individual midwives are put in that position – where is the midwife's primary obligation? To her patient or to society?*

(Interview 15)

The interview data does not provide precise information on what practices HCPs in Ireland withdraw from. However, participants' personal and professional reflections suggest that the willingness of HCPs to engage in conversations about abortion is impacted by ‘fear of future’. One interviewee stated that there was a culture of “don't ask, don't tell” around abortion (Interview 15); another felt that she had to wave as patients walked away rather than give them accurate information about abortion services (Interview 22). This, participants argued, was compounded by concerns about covert surveillance with some stating that “you have to be careful who is in the room…that you are not being recorded” (Interview 25). Importantly, such comments reinforce the fact that the practices of HCPs were conditioned by fear rather than what law or policy actually said. The provision of information about abortion services is not prohibited in either jurisdiction (in the Republic this right is contingent on the contemporaneous provision of information about continuation of a pregnancy and adoption). The law as written does not necessitate the self-censoring described by interviewees.

However, fear of imagined futures did not solely exert a regulatory effect. Analysis of interview transcripts also points to creative thinking by HCPs in response to fears about future ramifications. For example, while interview data indicates that HCPs regulate how and when they provide information about abortion services in response to what might happen if they are later judged as having broken the law, they also experiment with how information *can* be provided. As one interviewee described:

*We are kind of – if you write a letter of referral for a termination you are breaking the law. Write a letter of referral – ‘could you give me a second opinion’ – you are not breaking the law.*

(Interview 24)

Specialists at sexual and reproductive health (SRH) HCPs also argued that one tactic adopted by colleagues in hospitals - who were fearful of future judgement as having illegally offered abortion service information - was to direct women towards specialist family planning and SRH clinics (whose role explicitly included the provision of information about abortion). By suggesting that patients approach specialist SRH centres, HCPs could ensure that women would receive accurate information and at the same time minimise the potential for future judgement as having acted outside legal restrictions.

### Fears of abortion seekers' future

In addition to fear of personal futures, qualitative data revealed HCPs' fears of what may happen to abortion seekers in the future. For example, participants' in the study expressed fears of whether women would receive appropriate care. As one obstetrician explained, as women living in Ireland were leaving the Irish care system, Irish health professionals could have no control over or input into the care these women receive. This interviewee found this wholly problematic as there was no way for her to guarantee that her patients were provided with appropriate care or make sure that the abortion care received “did not harm or hurt her” (Interview 22, group interview). This lack of control over the care-giving process was raised in sharper terms by another obstetrician in the same interview. This interviewee stated that, while there were some centres in England Irish health practitioners could speak to about care for women travelling on an informal basis, if women travelled to other countries they “don't know where they go” (Interview 22, group interview). This interviewee stated that such lack of knowledge made post-abortion care challenging as practitioners could not be completely certain of the quality of care received at the clinic. While these interviewees expressed their concerns through the language of control and co-governance, underpinning their statements is a fear of the potential care women will receive in other care centres.

Data from interviews does not only point to HCPs' fearfulness of abortion seekers receiving or accessing inappropriate or inadequate care in the future. The potential for abortion seekers to be mistreated by ‘rogue’ agencies once they left their care was an acute source of fear for HCPs in Northern Ireland and the Republic. Rogue agencies – unregistered or unregulated SRH centres and clinics - are a significant problem in Irish abortion and reproductive health care. A series of media exposés in 2017 led by the Irish Times newspaper found that rogue and unregistered family planning clinics in the Republic of Ireland were providing women with misinformation. This was also reported by participants in the LIAC study with interviewees in Northern Ireland and the Republic of Ireland describing instances of women being given factually inaccurate information or led to unregistered clinics. One midwife interviewed provided a personal account of how a woman she encountered:

*was referred to Marie Stopes, went up there, and then outside of Marie Stopes was, sort of, fished for these – you know there are these rogue crisis pregnancy places? So they were outside of Marie Stopes, gave her a leaflet about “Oh we will ring you for a free scan, we will take care of you and all”. So they brought her for free scanning and they minded her and they were like “Oh we'll give you counselling, we'll give your husband counselling” – she had two kids already, she was from an east Asian country or something – and “we will help you, we'll help you financially” and all that and basically just coaxed her back into having the pregnancy. In the end she miscarried. She came in to us and said “Oh I've been to” and she named this place and free-scanning, what? And gave these two scans, they hadn't put in a letter of referral just two scan pictures. And she came in miscarrying. And you know she was obviously quite open to that sort of form of convincing. Like “we can help you this way and this way”. Whereas for other people it is going to be what it is – super manipulative. But that can bring people to a very bad place and I know the stuff that they tell people is not factual.*

(Interview 22)

Importantly it was not just the potential for abortion seekers to be taken in by these agencies that this interviewee was afraid of but the fact that HCPs could not protect women from these due to the illegality of referral. She felt that she could not control “what's going to happen to [abortion seekers] afterwards” and could only “wish [women] the best as they walked away” (Interview 21).

Participants in the LIAC study were also concerned about the *willingness* of women to access post-abortion services and fearful of the impact of this future unwillingness on post-abortion health and wellbeing. Here two key points were raised by participants – patient's future perception of their actions as illegal and their emotional responses to having had to travel for abortion services. The perception of illegality emerged during discussions about disclosures of information by women who had had abortions. A number of interviewees spoke of women not disclosing essential information regarding their treatment – or, in some cases, whether they had had an abortion at all – either because they were uncertain whether they had committed a crime or they were unsure about HCPs' reaction. For example, one interviewee, a midwife in an emergency room in the Republic of Ireland, described how she had encountered women who had delayed treatment because of this uncertainty:

*Like, I've had a women come in, you know, bleeding so much and she had delayed coming into see us and she was asking me ‘am I going to get in trouble?’*

(Interview 21)

Another interviewee, a crisis pregnancy counsellor at a family planning centre in the Republic of Ireland, also described how some women seeking information on or who opt for an abortion will ask “is it ok for me to be here?” (Interview 25).

The future emotional experiences of abortion seekers were also raised as concerning by interviewees. The effect of both having to travel for abortions or being refused abortion care was raised by a number of interviewees. HCPs participating in the study expressed concerns about future “complex bereavement patterns” (Interview 22), “trauma” (Interview 25), and feelings of abandonment (Interviews 15, 17, 23, 26). Interviewees also spoke at length on future self-stigmatisation by women who had had abortions and the effect this could have on their future emotional wellbeing.

Interestingly in terms of their influence on health providers' actions, the result of fear about abortion seekers has been the emergence of more creative ways of working and adaptations to mitigate problems prophesised in interviews rather than withdrawal from engaging in particular practices. For example, to ensure that care appropriate to abortion seekers' health needs is available at receiving centres, HCPs in Ireland have developed relationships with clinics abroad to facilitate the transferral of patient notes. As one interviewee described:

*We had [contact persons at a receiving clinic in England] and our doctor's had direct lines to them. They could explain the medical history, gestation and then ask if they could see her. Then they could get appointments booked, it's much more expensive to be treated as a private patient in a hospital than it is to be treated in a clinic. It meant the woman was sure when she left us, she had a date to go, she knew who she was going to see, she knew the cost – all she had to do was book her flights and accommodation.*

(Interview 17)

Similarly, another interviewee described how she and her colleague had developed a list of contact information for counterparts in receiving clinics which they would informally recommend to abortion seekers as “good places to go” (Interview 23). These details were distributed to colleagues at their hospital to make “very clear for everyone to see that you ring this number at this time, you fax over this amount of documentation” (Interview 23). Another interviewee (Interview 21) stated that she had approached her manager about highlighting their post-abortion care services on their hospitals' website as a way of encouraging abortion seekers' to access post-abortion care. Other HCPs described how they worked to encourage abortion seekers' to come back for post-abortion care so that patients did not feel isolated and to mitigate future self-stigmatisation. Staff at a foetal medicine unit, for example, spoke of the relationships they tried to foster with patients who had to travel for abortion services and how they made sure to arrange post-abortion check-up appointments and meetings with the women before they travelled for the procedure.

These accounts are noteworthy as they illustrate how fear resulting from an imagined future does not only regulate HCPs, it also encourages reflection on how care can be provided within the present legal and political context. The upshot of this has been the emergence of more creative ways of working.

## Conclusions

The objective of this article was to interrogate HCPs activities/inactivity in the context of abortion care. As we noted in the introduction, although current law and policy's curtailment of HCPs' ability to provide abortion care is obvious, their ‘on the ground’ interactions and navigation of the law - how they care, what they offer, and why - is more opaque. As De Zordo and others highlight, HCPs make decisions on what practices to engage in or not based on issues unrelated to what legal frameworks say on a daily basis. Liberalisation of abortion access in law has not always proven to equate to liberalisation of abortion access in practice. In the Irish context, the opacity of HCPs' logics of what care to offer/not offer is not helped by the fact that, outside of a very small number of studies (Aitken, Patek, & Murphy, 2017; Britton et al., 2017; Francome, 1994), qualitative evidence of the lived experience of HCPs caring for women is largely absent in existing literature.

What distinguishes this article is its proposal that the actions - or inactions - of HCPs are not just driven by what is permissible under the law or their moral position on abortion. Their fears for their own futures and those of their patients are equally significant. Analysed through the lens of affect and governing, research with HCPs conducted as part of the LIAC study illustrates that HCPs reasons for not engaging in certain forms of care-giving is not solely because they are legally restricted but because of what they imagine will happen their engagement with these practices be positioned as unlawful *in the future*. This is a much more complex dynamic than a straightforward reaction to coercive regulation or conscientious objection. Decision-making is affected by an imagined – but unrealised – futurity.

However, the application of affect theory is not just an intellectual exercise. It offers a starting point for a more overt discussion on what happens after restrictive abortion law and policy is liberalised. By rooting the reasons for caring/not caring in fear of the future which is entangled with law but not necessarily what the law *says*, this assessment encourages us to think about whether HCPs actions will automatically dramatically change if and when abortion laws in Ireland are altered. As the findings from the LIAC study indicate, what directed HCPs actions is not necessarily their understanding of these actions as legal/illegal but the likelihood of these actions becoming reframed as illegal at a future point. A common concern voiced by LIAC participants was that, even if they acted in accordance with the law, their interactions could be recorded without their knowledge (Interview 22, 25) or reported afterwards (Interview 15) as illegal and they could later find themselves facing criminal charges. Following affect theory writing, what we *imagine* the future to contain - Deleuze's Body without Organs (Bignall, 2010) or Ahmed's ‘hap’ (Ahmed, 2010) - has a much greater influence over our behaviour than what we *know* in the present. As abortion law in Ireland is renegotiated, it is important to think about the operationalisation of law and the experience of those practising within it. The perspectives, professional experiences, and realities of HCPs need to be included in the design and implementation of abortion law. The production of training and guidance for HCPs on the implications of the abortion law is also essential to avoiding HCPs over-regulating their work.

An affective reading of HCPs' fears is not only significant because it raises this question mark over whether care will become more freely available under a liberal abortion law. It is also important because it broadens the discussion on HCPs' reaction to restrictive abortion law and policy away beyond the practices they *withdraw* from (what they do not do) and includes the practices they *create* (what they do do). As Massumi (2002) emphasises, the sensations evoked by the imagining of the future in the present do not propel action in a specific direction. The moment when the present subject becomes aware - either physically or through the imagination - of the ‘not yet’ is a creative space. The possibilities for new ways of acting to emerge from this moment are, for affect theorists, limitless (Gregg & Seigworth, 2010). Admittedly, developing dialogues with abortion providers outside Ireland in order to transfer patient notes is not wholly revolutionary. However, the point is that the emotional reaction to future thinking on the part of HCPs (fear) does not only lead to them restricting the care they provide but to experiments in how to deliver care.

These arguments are not just relevant to the Irish context. Globally and in Ireland, debates on abortion law and policy and the availability of abortion ‘on the ground’ are subject to increasing scrutiny. Crucially, these debates do not just focus on areas where abortion is illegal. Supposedly liberal legal regimes struggle with the translation of the theoretical accessibility of legal abortion into a health care reality. De Zordo and Mishtal (2011) and others (Purcell et al., 2017; Beynon-Jones, 2013; Hoggart, 2015; Harris, Debbink, Martin, & Hassinger, 2011; Norris et al., 2011) have written at length about HCPs withdrawal from abortion practice due to issues outside of legal frameworks. Personal feelings of moral opposition and stigma all play a role in what care is offered, under what circumstances, and in what ways. Outside of academic writing, abortion rights advocates and SRH agencies globally have highlighted that abortion is being refused even in areas where it is legally permissible (Centre for Reproductive Rights, 2018; International Planned Parenthood Federation, 2016). HCP disengagement is emphasised as problematic by International Planned Parenthood Federation and the World Health Organisation. Focused discussions and analyses of why HCPs are reticent to provide abortion care is useful to these debates.

In combination with literature on conscientious objection, stigma, and regulation, the affective reading of HCPs' activity/inactivity presented in this article illustrates the complexity of decision-making and care-provision at the front line. Given the substantial health implications of HCPs' fearfulness of what will happen if they do or do not care for women, the insights offered by the LIAC study indicate that the emotional registers of HCPs in abortion care require further analysis and discussion.

## Funding source

This work was supported by a Wellcome Trust Seed Grant in Humanities and Social Science [Grant reference: 110469/A/15/Z].
